# Bacterial Metabolites and Inflammatory Skin Diseases

**DOI:** 10.3390/metabo13080952

**Published:** 2023-08-17

**Authors:** Victoria Jiminez, Nabiha Yusuf

**Affiliations:** 1 Heersink School of Medicine, University of Alabama at Birmingham, Birmingham, AL 35294, USA; vjiminez@uab.edu; 2 Department of Dermatology, University of Alabama at Birmingham, Birmingham, AL 35294, USA

**Keywords:** gut-skin axis, bacterial metabolites, microbiome, inflammatory skin disease, atopic dermatitis, psoriasis, hidradenitis suppurativa, immune regulation

## Abstract

The microbiome and gut-skin axis are popular areas of interest in recent years concerning inflammatory skin diseases. While many bacterial species have been associated with commensalism of both the skin and gastrointestinal tract in certain disease states, less is known about specific bacterial metabolites that regulate host pathways and contribute to inflammation. Some of these metabolites include short chain fatty acids, amine, and tryptophan derivatives, and more that when dysregulated, have deleterious effects on cutaneous disease burden. This review aims to summarize the knowledge of wealth surrounding bacterial metabolites of the skin and gut and their role in immune homeostasis in inflammatory skin diseases such as atopic dermatitis, psoriasis, and hidradenitis suppurativa.

## 1. Introduction

Commensal microbiota of the skin and gastrointestinal (GI) system have been linked to the homeostasis and pathogenesis of inflammatory skin diseases. Specific bacterial species have been shown to regulate pro- and anti-inflammatory responses in the skin. The interplay between the microbiome and skin inflammation has been associated with conditions such as atopic dermatitis, psoriasis, connective tissue diseases, and other autoimmune inflammatory disorders such as lupus erythematosus [1]. Relationships between the immune system and these inflammatory skin conditions have been well studied. However, the influence of bacterial metabolites on immune regulation has only begun to be investigated for its subsequent application to skin inflammation. The main metabolites associated with immune physiology include short-chain fatty acids (SCFAs), tryptophan metabolites, and amine derivatives such as trimethylamine N-oxide (TMAO) [1,2,3,4]. The gut-skin axis has become a topic of increasing interest in recent years due to the ability of next-generation sequencing to characterize microbial compositions as well as the discovery of concomitant gut alterations in those with skin disorders [5,6,7]. Disruption of this relationship and its implications on the pathophysiology of inflammatory skin conditions have led to questions of whether clinical interventions pose efficacy in re-establishing a homeostatic balance between the gut and skin [8]. Debate in this area of research currently discusses whether microbial dysbiosis is a causation or result of inflammatory skin diseases and whether certain metabolites play different roles in the skin and GI tract.

This review serves to provide a concise summary of recent literature regarding specific bacterial metabolites of both the skin and gastrointestinal tract implicated in inflammatory skin conditions such as atopic dermatitis, psoriasis, hidradenitis suppurativa, and others.

## 2. Discussion

### 2.1. Cutaneous Microbiome in Atopic Dermatitis

The pathogenesis of atopic dermatitis (AD) is multifactorial, including aberrant immune responses, skin barrier defects, and environmental allergen and microbe effects. Environmental cutaneous exposures have been well-established in this dynamic disease-causing eczema flares and superimposed infections [9]. The cutaneous immune response is essential due to the increased trans-epidermal water loss seen in AD skin; however, the gut immune response has yet to be as thoroughly elucidated in its effects [10]. This increased susceptibility to infection that affects 15–20% of children puts this population at a significantly higher risk of developing skin and soft tissue infections, eczema herpeticum, bacteremia, osteomyelitis, septic arthritis, and endocarditis [11,12]. Up to 90% of people with AD are heavily colonized with *Staphylococcus aureus*, and the enterotoxins (superantigens) produced by this bacteria may contribute to keratinocytes apoptosis, skin barrier defects, and mast cell degranulation [9,10,13]. Cutaneous barrier dysfunction via genetic and environmental factors contributing to AD allows for aberrant alteration of commensal bacteria. Recent literature on the AD skin microbiome reveals increased density of lesional AD skin overall with increased relative abundance and burden of *S. aureus*. Microbiome analysis of eczematous lesions in mice has revealed prominent dysbiosis similar to that of humans, with skewed prominence of *S. aureus* and *Corynebacterium* spp. [14]. Numerous studies have demonstrated dysbiosis of the skin microbiota in AD, as evidenced by Alam et al.’s 2022 review on microbiota manipulation for AD treatment [15].

In comparison to the gut microbiota, skin microbiota is thought to have different metabolic functions, be nutrient-poor, and be more acidic in nature [16]. The two are often compared as part of the body’s overall microbiome, but play very different roles in terms of environment and immune regulation. The stratum corneum contains amino acids derived from keratin and dead keratinocytes that are thought to provide substrates for tryptophan (Trp) metabolism by the skin microbiota [16,17]. Tryptophan acts as an energy source and immunomodulator through other metabolites, such as indoles and their derivatives [18]. These indole derivatives mediate interactions between *Escherichia coli* and hosts and serve to tighten gut epithelial junctions [16,19]. Chng et al. conducted a whole metagenome analysis of 80 skim microbiome samples to reveal insight into how the skin surface microenvironment and immune system cross-modulate each other and found the tryptophan metabolic pathway to be attenuated in the skin microbiota of patients with AD [16,20]. A subsequent study by Yu et al. revealed that indole-3-aldehyde (IAId), a skin microbiota-derived Trp catabolite, negatively regulates skin inflammation in patients with AD [16]. These Trp derivatives act as ligands for aryl hydrocarbon receptors (AhR) that have been suggested to play an essential role in many physiological and pathological processes in the skin [21,22]. Activation of this AhR signaling pathway in epidermal keratinocytes initiates inflammatory skin lesions and has been implicated in inflammatory diseases such as psoriasis and AD [21,23]. With the findings from Yu et al. regarding IAId, it is thought that perhaps its stimulation of AhR may interact with pro-inflammatory thymic stromal lymphopoietin (TSLP) promoter regions in keratinocytes to suppress inflammation and promote immune homeostasis in the skin of healthy subjects [16,21].

It has also been suggested that AhR expression levels in peripheral blood mononuclear cells are higher than in AD patients and may be associated with eczema area and severity index scores [21,24]. Investigations into this pathway prompt the notion that IAId and tryptophan derivatives positively suppress inflammatory responses associated with AhR activation in AD. Additionally, Liu et al. investigated the role of Langerhans cells and their response to microbial metabolites of the skin [25]. They found that IAId acted as a negative regulator on LCs by promoting activation of AhR and IL10 production and inhibition of LC-induced CD4+ T cell proliferation. Through this pathway, these tolerogenic properties of LCs may be utilized for future treatment of inflammatory skin diseases [25]. According to other studies, some AhR ligands activate the antioxidative transcription factor Nrf2, attenuating inflammatory responses in AD and psoriasis [21,26,27]. In this case, the use of AhR agonists such as tapinarof have confirmed efficacy in clinical studies for AD [21,28]. While it is evident that the AhR pathway is implicated in AD pathogenesis, further delineation is needed to address which ligands serve as optimal regulators of anti-inflammation and whether therapeutic targets related to tryptophan and indole-related derivatives exist.

**Figure 1:**
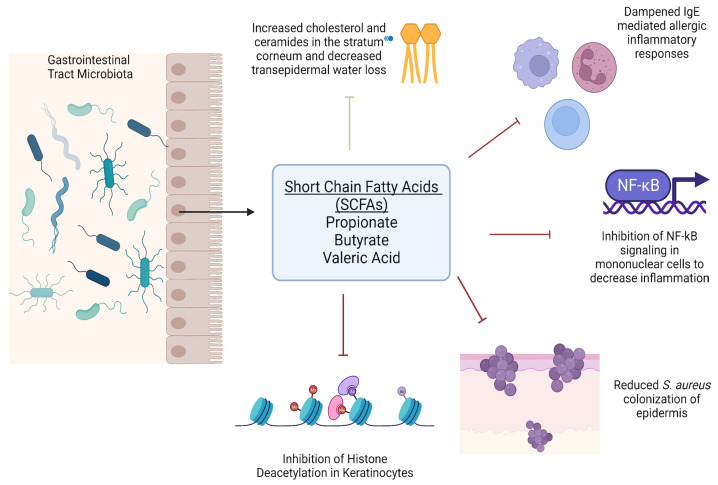
Roles of short chain fatty acids on immune function and epidermal homeostasis (Created with Biorender 2023 version).

In atopic dermatitis lesions, *S. aureus* has also been found to co-exist with other commensal bacteria on the skin surface, such as *Cutibacterium acnes (C. acnes)* and *Staphylococcus epidermidis (S. epidermidis)* [29,30]. Fermentation products of carbohydrates such as short-chain fatty acids such as propionic acid are produced by *C. acnes* and have been shown to have anti-inflammatory activities and regulate the production of cytokines such as TNF-a, IL-2, IL-6, and IL-10 [29,31]. *S. epidermidis* also ferments glycerol to butyric acid and acetic acid that suppress the growth of methicillin-resistant *S. aureus* infections [29]. Butyric acid has functions, including inhibiting histone deacetylases in keratinocytes to suppress inflammation and attenuate lipopolysaccharide-induced NFkB activation and nitric oxide production [29,32,33]. SCFAs also regulate the ability of immune cells to migrate towards inflammatory loci in AD lesions [34]. Recently, Traisaeng et al. demonstrated that *S. epidermidis* could mediate glycerol fermentation to reduce skin colonization by AD *S. aureus* and that high concentrations of butyric acid can kill AD *S. aureus*. Their previous data also showed propionic acid effectively killed MRSA strains by reducing intracellular pH [29,35]. Additionally, a 2023 comprehensive review by Stec et al. concerning gut microbiota and dermatological diseases consolidated data from multiple studies that associate AD dysbiosis with low butyrate and propionate levels and the bacteria that produce them, as well as the finding that higher butyrate levels correlated with less severe disease [1,36,37,38,39,40]. Dysregulated fecal SCFAs and valeric acid production were also associated with a higher prevalence of disordered microbiota and AD development risk [1,39,41,42]. These findings lend interest to the possibility of optimizing butyric acid metabolite concentrations via therapeutics. SCFAs also act to dampen IgE allergic responses, influence cholesterol and ceramide concentrations, and decrease transepidermal water loss for the maintenance of the epidermal barrier in AD and other inflammatory skin diseases [1]. A depiction of their functions can be seen in Figure 1.

The microbial makeup of the skin is strongly influenced by intraspecies competition and antimicrobial peptide production by both the host and competitive strains of bacteria [43]. Commensal populations of coagulase-negative staphylococci (CoNS) can inhibit nonresident pathogenic bacteria such as *S. aureus*, group A. streptococci, and *Escherichia coli* through the production of bacteriocins [43,44,45,46,47]. Small cyclic peptides, known as autoinducing peptides (AIPs), are also produced by CoNS to kill *S. aureus*, specifically [43,48,49]. However, most patients with AD lack protective strains of CoNS in addition to deficiencies in AMPs. Nakatsuji et al. isolated a strain of *Staphylococcus hominis* significantly lower in those with AD vs. healthy adults and conducted a phase I randomized clinical trial with its use topically [43]. This method of bacteriotherapy sought to reestablish the skin commensal microbiome by replenishing protective microbes and their metabolites against damaging *S. aureus*. The primary endpoint of safety was met, and although eczema severity was not significantly different, secondary endpoints were met for significant decreases in *S. aureus* were seen [43]. Improvement in local eczema severity was suggested by post-hoc analysis and lend hope towards the future success of targeted microbiome transplant for AD [43].

While *S. aureus* has been highly implicated as a negative modulator of AD exacerbation, some studies have observed overlap in areas of skin affected by AD and areas typically colonized with gram-negative bacteria in healthy controls, but not in AD patients [30,50,51,52]. This suggests the possibility of a homeostatic role of commensal gram-negative bacteria in the skin microbiome. Myles et al. discovered that the gram-negative bacteria *Roseomonas mucosa* isolated from healthy volunteers improved outcomes in AD mice and cell models. In contrast, AD-sourced *R. mucosa* had no impact or worsened outcomes [52]. In a phase I/II safety and activity placebo-controlled clinical trial for topical microbiome transplantation with *R. mucosa*, treatment was associated with significant decreases in subjective and objective measures of disease severity, topical steroid requirements, and *S. aureus* burden [52]. Follow-up studies showed skin improvements and colonization up to 8 months afterward. The mechanism for its efficacy was also further studied, suggesting that the production of sphingolipids by *R. mucosa* may have contributed to the therapeutic impact [53]. Sphingolipids and their downstream antimicrobial peptides are deficient in the skin of people with AD as the sphingolipid pathway is linked to the control of *S. aureus*, epithelial barrier maintenance, and immune regulation [53,54,55,56]. Subsequent studies on 14 patients prior to and after treatment with *R. mucosa* demonstrated increases in sphingomyelin and related lipids, suggesting alterations in arachidonic metabolism with treatment [53]. Further clinical trials are warranted to investigate the long-term efficacy and optimal therapeutic interventions for both *R. mucosa* and other gram-negative microbial commensal transplantations for AD. Subsequently, a biotherapy has been developed as a live bacterial formulation skin dressing with *R. mucosa*, hypothesized to colonize and restore the skin microbiome and suppress commensal *S. aureus* and inflammatory responses [57].

### 2.2. Gut Microbiome in Atopic Dermatitis

The gut microbiome has become an increasing area of popularity in studying its modulatory effects on systemic inflammation, specifically inflammatory dermatoses [1,58]. One of the focused realms of study related to this topic is the use of probiotic bacterial metabolites for their positive effects on inflammatory suppression and homeostatic maintenance. These live microorganisms are thought to restore function in gut dysbiosis and stimulate the production of SCFA metabolites generated by anaerobic bacteria [59,60]. Regulation of the immune response by *Lactobacillus* spp. have gained attention for their strong ability to decrease Th1, Th2, and Th17-related cytokines and increase IL-10 and CD4+CD25+ regulatory T cells [60,61,62,63,64]. It has been shown to alleviate AD via modulation of gut microbiota [63]. A study by Kim et al. showed that with the administration of *Lactobacillus fermentum* in AD-induced mice, significant reductions in serum IgE, tissue mast cells and eosinophils, and Th2 related cytokines, with increases in anti-inflammatory cytokines IL-10 and transforming growth factor-B [60]. Metabolic analysis of the cecum showed significant changes in treated mice in levels of amino acids, including methionine, phenylalanine, serine, and tyrosine, and SCFAs such as acetate, butyrate, and propionate [60]. Further, Matsumoto et al. also investigated the effects of probiotics with the administration of *Bifidobacterium animalis* in yogurt. This double-blind, placebo-controlled crossover study found that scores of itch and burning improved to a greater extent, IFN-y serum levels significantly increased, and fecal spermidine and butyrate concentrations significantly increased [65,66]. The aforementioned comprehensive review of microbial manipulation by Alam et al. consolidated all recent studies regarding probiotic administration with further efficacy surrounding *Lactobacillus* spp. [15,67,68,69,70]. A recent metanalysis of randomized control trials found that intake of *L. rhamnosus* during pregnancy significantly lowered the risk of infantile development of AD at 2 and 6–7 years of life [1,71]. The findings from these studies suggest the plausibility of gut microbiome alteration to produce these various metabolites through probiotic therapeutics.

Further, fecal microbiota transplantation (FMT) has even been suggested as a potential new therapy for AD. A recent study aimed to restore gut microbiota in AD mice via FMT to ameliorate AD-induced allergic responses [72]. Gut metabolite levels were determined by fecal SCFA contents and increased post-FMT [72]. FMT also restored the balance of Th1/Th2, modulated T-regs, reduced IgE levels, and the number of mast cells, eosinophils, and basophils, suggesting suppression of AD immune responses [72]. Conclusions from this study suggested FMT may be more effective than probiotics for long-term efficacy of restoring gut dysbiosis and subsequent AD treatment. However, it should be kept in mind that the risks of FMT are not well known, and those with existing gut dysbiosis may have a more compromised barrier and be more susceptible to these risks [15]. While many inflammatory pathways and cytokines are well established in AD pathogenesis and regulation, Hou et al. demonstrate that there is more to be discovered through the cytokine IL-37b of the IL-1 family [73,74,75]. In IL-37b knock-in mice, this cytokine showed a distinct intestinal microbiota pattern and restored gut microbiota diversity [73]. This occurred via regulation of the in vivo autophagy mechanism mediated by intestinal metabolite 3-methyladenine, adenosine monophosphate, 2-hydroxyglutarate, purine, and melatonin, suggesting IL-37b as a potential anti-inflammatory cytokine for AD treatment [73]. Human models and further delineation of ways to upregulate this cytokine in the gut of those with AD is needed. A recent article published in March of 2023 brought to light considerations of the gut virome and its effects on bacterial metabolism in the context of the entire microbial environment, suggesting a possible mechanism for bacterial phage contributions to overall gut health and skin health [76]. They observed fecal samples in a 2-year-old boy over six months and found temporal correlations among virome alterations, microbial metabolite changes such as downregulation in the catabolism of aromatic amino acids, and symptom remission [76].

Overall, it has become apparent from recent literature that both cutaneous and gut commensal microbiota play a significant role in AD disease pathogenesis. Alterations of specific flora populations contribute to overall immune homeostasis of the skin. With variations in microbial makeup, subsequent production and signaling of bacterial metabolites are also altered and affect disease development and exacerbations systemically and on the local level. Future studies are indicated to optimize the production of anti-inflammatory bacterial metabolites by maximizing colonization of beneficial commensal microbes and minimizing aberrant *S. aureus* growth in AD susceptible skin areas. A summary of key studies related to specific metabolites associated with AD can be found in Table 1.

### 2.3. Skin Microbiome in Psoriasis

Psoriasis is another inflammatory skin disease with multiple subtypes and heavily immune-mediated pathogenesis with a predominant IL-23/Th17 axis [77]. Inflammation of the skin and joints is the most common presentation, but it has been identified as a systemic entity due to its associated comorbidities. These include an increased risk of developing hyperlipidemia, coronary artery disease, and type 2 diabetes compared to controls [77,78]. In addition to metabolic syndrome, psoriasis has been associated with inflammatory bowel disease. It has also been postulated that nutrition and diet influence psoriatic patients in that saturated fatty acids, simple sugars, red meat, and alcohol are thought to exacerbate disease through many immune mechanisms and gut dysbiosis [79]. Roles have also been suggested for vitamins D and B12, SCFAs, genistein, selenium, and probiotics to ameliorate psoriasis or its comorbidities [79].

SCFAs such as butyrate that were heavily discussed surrounding AD also play a role in psoriasis via their induction of differentiation of thymic T regulatory cells and naïve CD4+ T cells into peripheral Tregs by histone deacetylase inhibition [79,80,81]. T regs in psoriasis patients have been reported to have reduced suppressive activity that normalizes with sodium butyrate administration and IL-10 levels and expression of Foxp3, IL-17, and IL-6 in psoriatic skin lesions [82,83]. Sodium butyrate also enhances keratinocyte differentiation and mRNA of filaggrin and transglutaminase A, while promoting cornified envelope formation of keratinocytes and downregulating their proliferation [82,84]. SCFAs can be produced by *Cutibacterium acnes* for homeostasis and act via G-protein-coupled receptors (GPCRs) [79,85]. Certain GPCRs in psoriatic skin have decreased expression compared to control skin, suggesting perhaps an absence of normal SCFAs produced by commensals as well as a reduced ability to respond to them in psoriatic skin [79,86]. Absence of beneficial bacterial phyla have been implicated as potential mediators of dysregulation and inflammation in the skin and joints. The AhR tryptophan signaling pathway has been implicated in psoriasis, much like in AD [21,23]. AhR-mediated Th17 activity upregulates the production of IL-22, a cytokine that contributes to the increased proliferation of epidermal cells and whose plasma concentrations have been correlated with disease severity [21,87,88,89,90,91]. 6-formylindolo[3,2-b]carbazole (FICZ) is a ligand of AhR that is known to reduce inflammatory responses in skin lesions and psoriasis [21,92]. It has been suggested that the microbiome may modulate some properties of AhR signaling, and optimal therapeutics or interventions such as FICZ regulation and tryptophan dysregulation require further investigation [21]. AhR agonists have ameliorated imiquimod-induced psoriasis in mouse models and may be a therapeutic target [17].

### 2.4. Gut Microbiome in Psoriasis

Intestinal overrepresentation of *Escherichia coli*, *Salmonella*, *Campylobacter*, *Helicobacter*, *Alicaligenes*, and *Mycobacterium* species has been observed in psoriasis as well as an increased *Firmicutes-to-Bacteroidetes* ratio (*F*/*b)* [93]. Studies have shown decreased intestinal microbiome diversity in psoriasis patients compared to controls and reduced beneficial microbiota such as *Parabacteroides*, *Coprobacillus*, and *Faecalibacterium prausnitzii* [79,94,95]. These are known bacteria to produce SCFAs, and Olejniczak-Staruch et al. concluded from previous studies that intestinal dysbiosis in psoriasis and psoriatic arthritis is characterized primarily by lower production of butyrate due to this dysbiosis [94]. In turn, the intestinal barrier is weakened and becomes more susceptible to systemic bacterial-induced inflammation and the formation of psoriatic phenotypes. While there have been reports of no difference in SCFA fecal concentrations in psoriasis, others confirm reductions in concentrations of enzymes involved in the synthesis of butyrates and decreased abundances of these SCFA-producing bacteria [93,94,96]. Fecal samples have also revealed reduced expression of receptor activator of nuclear factor kappa-B ligand (RANKL) that may be due to bacteria typical of psoriasis and psoriatic arthritis or indicate the modulating effect of this molecule on systemic inflammation [93,94].

There has been discussion in the literature surrounding an association between multiple inflammatory diseases such as psoriasis and trimethylamine oxide (TMAO), a molecule involved in cholesterol and cardiovascular disease processes [93,97,98]. This gut metabolite has been associated with the elevated *F*/*b* ratio seen in psoriasis and is produced by bacteria capable of metabolizing carnitine to TMA [93]. This associated increase in *F*/*b* ratio may result in the limitation of SCFA and butyrate production and may predispose people with psoriasis to the development of metabolic syndrome [93,99,100]. Correlations between other bacterial species such as *Vibrio*, *Ferruginibacter*, *Romboustia*, and psoriasis have also been drawn in addition to specific metabolites [101]. Chen et al. showed a significant positive association of psoriasis with itaconic acid, crotonic acid, and heptadecanoic acid, all involved in lipid metabolism. Negative associations were also made with several lipids, xanthine, d-ribose 5-phosphate, and uric acid, suggesting a role of skin microbial influence on lipid and nucleotide metabolism [101]. Conclusions of this study include evidence for underlying mechanisms of skin microbiome-mediated regulation of blood and lipid metabolism in addition to inflammatory responses in psoriasis patients [101]. People with psoriasis and concomitant metabolic syndrome have also been observed to have higher levels of lipopolysaccharide-binding protein (LBP), an indicator of serum LPS, a toxic bacterial byproduct [102]. With the known associations of immune dysregulation and epidermal proliferation in psoriasis, identification of the most prominent metabolite contributors will be essential to the understanding of comorbidities and future development of therapeutics and interventions to manipulate disease state in this patient population.

### 2.5. Micriobiota in Additional Inflammatory Cutaneous Conditions

While AD and psoriasis are the inflammatory dermatoses that have been more extensively investigated in their pathogenesis related to bacterial metabolites, there have been studies related to the subject in hidradenitis suppurativa (HS), acne, and rosacea as well. Crohn’s disease has been reported as being the most associated disease with HS [103]. A pooled data analysis even suggested a prevalence of HS in IBD patients of 12.8% [103]. While both genetic and environmental factors such as smoking have been suggested as associations, recent findings look towards an interplay between intestinal and skin microbiota. HS lesions have an abundance of *S. aureus* and coagulase-negative staphylococci, although different cutaneous regions have been seen to have different microbial communities [104,105]. One study has even shown the presence of *S. epidermidis* biofilms present in hair follicles and sinus tracts [103,106]. It has been hypothesized that unregulated inflammation may cause lesions typical of both HS and IBD, which need further exploration in terms of specific metabolites that may relate to the pathogenesis of immune dysregulation and disease exacerbation in both [103]. While depletion of *Faecalibacterium prausnitzii* was seen in psoriasis patients, when studied in HS, its relative abundance was only decreased in cases where patients had concomitant IBD and HS [95].

Trimethylamine oxide (TMAO) has also been investigated as a bacterial metabolite in HS. Barrea et al. found increased circulatory TMAO levels in HS patients and correlations of these levels with increased HS Sartorius scores after adjustment for body mass index and waist circumference [107,108]. Those with more severe Hurley stage II disease also had higher TMAO levels compared to stage I [107,108]. While no studies have explored toxins such as LPS in HS, increased levels of LBP in psoriasis and obesity may also be present in excess in HS [107,109,110]. Luck et al. hypothesize that perhaps bacterial pathways leading to the production of harmful metabolites may be associated with or contribute to HS development in addition to microbial dysbiosis [107]. The exact pathogenesis of HS is still up for debate, but is proposed to be multifactorial with infectious components. In a summary of HS research during the last 15 years in the European Hidradenitis Suppurativa Foundation official journal, authors found downregulation of alarmins/antimicrobial peptides of S100A and S100A9 and increased expression of antimicrobial cathelicidins LL-37 in HS lesional skin, suggesting innate barrier dysfunction and development of altered host-microbiome crosstalk [111]. Additionally, one of the theories related to smoking-induced HS exacerbation is the finding that nicotine promotes the growth of *S. aureus* and alters the microbiome and synthesis of antimicrobial peptides such as hbD2, rendering hair follicles weaker against pathogens [111].

Alterations in metabolic pathways by bacterial microbes include those involved with amino acids, carbohydrates, and lipids in HS [112,113]. Schell et al. recognized a consensus from multiple studies revealing increased abundances of anaerobic bacteria and opportunistic pathogens that seem to replace normal commensals such as *Cutibacterium* in HS skin [112,113,114,115,116,117]. Multiple metabolite synthesis pathways are dysregulated in HS including ATP and extracellular nucleotide receptors, the NLRP3 inflammasome, and the production of specific cytokines [113,118]. Amino acid and tryptophan sensing are also altered, including the aryl hydrocarbon receptor, as seen in both AD and psoriasis [113,119]. Tryptophan is aberrantly catabolized into kynurenine, and catabolism into indole metabolites is reduced, which in turn reduces AhR activation that may drive inflammation in HS due to microbiome dysbiosis [113,119]. Mass spectrometry has also identified increased levels of SCFAs in HS skin, which is contradictory to the decreased presence of SCFAs and butyrate in AD and psoriasis, but SCFAs have limited study in HS pathophysiology [113,120].

Acne is another skin inflammation disease where SCFAs produced by *C. acnes* on the skin in hypoxic, lipid rich conditions have been reported to have a pro-inflammatory effect on epidermal keratinocytes [121,122]. Keratinocytes treated with SCFAs also have shown increased proinflammatory cytokine responses. This effect contrasts the well-established anti-inflammatory effects of SCFAs on cells of myeloid origins and the reported benefits of SCFAs in the gut microbiome in other inflammatory dermatoses [121,123,124,125]. Sanford et al.’s 2019 study supported speculation that SCFAs from *P. acnes* drive cytokine expression prior to follicular skin rupture and may influence local pilosebaceous units and surrounding skin [121]. Special considerations must be taken for acne-specific skin sites that may contribute to the supposed contradictory effects of SCFAs in other inflammatory skin diseases. For example, acne-prone skin is characterized by high amounts of free fatty acids in sebum, such as lauric, palmitic, and oleic acids that induce antimicrobial peptide expression from sebocytes [121,126]. Authors note that the local follicular environment and its antimicrobial processes may be altered by fatty acids from the host, resident microbes, and their bacterial metabolites to induce inflammation [121]. Further investigation into types of SCFAs produced by various microorganisms and their effect on host responses is needed to characterize their deleterious effects further, if present.

The role of the gut and skin microbiome has even been discussed in the pathogenesis of rosacea. Polymicrobial commensalism of the skin involved in rosacea and sebum makes homeostasis of these areas essential to suppressing excess inflammation. Abnormal toll-like receptor signaling has been observed in rosacea, a group that often responds to specific microbial products and metabolites [127]. The epidermis of subjects affected by rosacea have higher TLR2 expression than healthy subjects, suggesting a mechanism for inflammatory signaling at a low threshold to external stimuli [127,128]. Triggers for TLR2 activation include bacterial products [129]. This enhanced expression can also cause the production of cathelicidin antimicrobial peptides and increased activity of serine protease kallikrein. Serine protease LKL-5 is involved in the cleavage of cathelicidin to the active peptide form L-37, a modulator of neutrophil chemotactic and stimulates cytokine and chemokine release from mast cells. These processes lead to erythema, angiogenesis, and telangiectasias seen in rosacea inflammation. Small intestinal bacterial overgrowth has also been associated with rosacea development, suggesting a systemic inflammatory process dependent on microbial communities and immune signaling [127,130].

## 3. Conclusions and Future Directions

The microbial environment of the skin and gut is vast in its diversity and role in immune signaling. With the abundance of data in recent years to characterize the makeup of specific microbial communities in inflammatory disease processes, the wealth of knowledge grows surrounding metabolite production and their functions in cutaneous and systemic inflammation. It is apparent that short chain fatty acid and aryl hydrocarbon receptor signaling pathways are essential to many metabolic and immune processes affected by inflammatory skin diseases. Future studies are warranted to identify states of optimal metabolite production for anti-inflammatory signaling and immune maintenance and whether those processes are varied between the cutaneous and gastrointestinal microbiome. With further confirmatory knowledge of these complex processes, the identification and use of therapeutics for microbial community modulation in the form of probiotics or immune modulators will make significant strides for future clinical application.

## Figures and Tables

**Table 1:** Summary of studies related to bacterial metabolites and their role in AD homeostasis.

Author	Metabolite or Microbe	Gut or Skin Microbiota	Findings/Conclusion
Chng et al. [20]	Tryptophan	Skin	Metagenome analysis revealed attenuation of Trp metabolic pathway in AD patients
Yu et al. [16]	Indole-3-aldehyde (Trp derivative)	Skin	Trp metabolites of skin microbiota play a significant functional role in AD and IAId induced AhR interactions may promote skin immune homeostasis
Liu et al. [25]	Indole-3-aldehyde (Trp derivative)	Skin	IAId activation of AhR in LCs inhibit CD4+ T cell proliferation and induce IL-10 production and immune tolerance
Nakatsuji et al. [43]	*Staphlococcus hominus*	Skin	Bacteriotherapy with CoNS may help restablish commensal bacterial metabolites to protect against *S. aureus*
Traisaeng et al. [29]	Butyric acid	Skin	Production of butyric acid derivatives by *S. epidermidis* inhibit growth of *S. aureus* in AD patients
Wang et al. [35]	Propionic acid	Skin	Propionic acid and its esterified derivative provide efficacy as antimicrobial agents against AD *S. aureus*
Myles et al. [52]	*R. mucosa* & sphingomyelins	Skin	Topical treatment was associated with amelioration of disease severity, improvement in barrier function, and reduction in both *S. aureus* and need for topical steroids
Myles et al. [53]	*R. mucosa* & sphingomyelins	Skin	Mouse models of AD found production of sphingolipids by *R. mucosa*, cholinergic signaling, and flaggelin expression may contribute to therapeutic impact in the previous trial [52]
Kim et al. [60]	*Lactobacillus* spp. for production of multiple metabolites	Gut	Administration of probiotic microorganisms reduced inflammatory immune responses associated with AD and increased levels of amino acids and SCFAs
Matsumoto et al. [65]	Fecal spermidine and butyrate	Gut	*Bifidobacterium animalis* in yogurt improved scores of itch and burning and significantly increased IFN-y serum levels
Kim et al. [72]	FMT	Gut	FMT resulted in an increase in levels of SCFAs as gut metabolites and decreases in blood parameters suggested of AD-induced allergic responses with suggested prolonged efficacy compared to probiotics
Hou et al. [73]	IL-37b cytokine	Gut	IL-37b restored gut dysbiosis in terms of diversity and could ameliorate eosinophil-mediated allergic inflammation via intestinal bacteria and metabolite modulation
Chu et al. [76]	Lytic crAssphage viral strains	Gut	Gut virome phage alterations manipulate gut bacterial production of aromatic amino acids and AD symptoms
